# Induction of Immunogenic Cell Death by Photodynamic Therapy Mediated by Aluminum-Phthalocyanine in Nanoemulsion

**DOI:** 10.3390/pharmaceutics14010196

**Published:** 2022-01-14

**Authors:** Mosar Corrêa Rodrigues, Wellington Tavares de Sousa Júnior, Thayná Mundim, Camilla Lepesqueur Costa Vale, Jaqueline Vaz de Oliveira, Rayane Ganassin, Thyago José Arruda Pacheco, José Athayde Vasconcelos Morais, João Paulo Figueiró Longo, Ricardo Bentes Azevedo, Luis Alexandre Muehlmann

**Affiliations:** 1 Laboratory of Nanoscience and Immunology, Faculty of Ceilandia, University of Brasilia, Brasilia 72220-900, Brazil; mosarcr@gmail.com (M.C.R.); jaquelinevazdeoliveira@gmail.com (J.V.d.O.); rayaneganassin@hotmail.com (R.G.); thyagojap@gmail.com (T.J.A.P.); joseathayde_9@hotmail.com (J.A.V.M.); 2 Department of Genetics and Morphology, Institute of Biological Sciences, University of Brasilia, Brasilia 70910-900, Brazil; wellingtonjunior123@hotmail.com (W.T.d.S.J.); thaynamundim@hotmail.com (T.M.); camilla_costavale@hotmail.com (C.L.C.V.); jplongo82@gmail.com (J.P.F.L.); razevedo@unb.br (R.B.A.)

**Keywords:** nanobiotechnology, apoptosis, damage associated molecules patterns, immunotherapy

## Abstract

Photodynamic therapy (PDT) has been clinically employed to treat mainly superficial cancer, such as basal cell carcinoma. This approach can eliminate tumors by direct cytotoxicity, tumor ischemia, or by triggering an immune response against tumor cells. Among the immune-related mechanisms of PDT, the induction of immunogenic cell death (ICD) in target cells is to be cited. ICD is an apoptosis modality distinguished by the emission of damage-associated molecular patterns (DAMP). Therefore, this study aimed to analyze the immunogenicity of CT26 and 4T1 treated with PDT mediated by aluminum-phthalocyanine in nanoemulsion (PDT-AlPc-NE). Different PDT-AlPc-NE protocols with varying doses of energy and AlPc concentrations were tested. The death mechanism and the emission of DAMPs–CRT, HSP70, HSP90, HMGB1, and IL-1β–were analyzed in cells treated in vitro with PDT. Then, the immunogenicity of these cells was assessed in an in vivo vaccination-challenge model with BALB/c mice. CT26 and 4T1 cells treated in vitro with PDT mediated by AlPc IC_50_ and a light dose of 25 J/cm^2^ exhibited the hallmarks of ICD, i.e., these cells died by apoptosis and exposed DAMPs. Mice injected with these IC_50_ PDT-treated cells showed, in comparison to the control, increased resistance to the development of tumors in a subsequent challenge with viable cells. Mice injected with 4T1 and CT26 cells treated with higher or lower concentrations of photosensitizer and light doses exhibited a significantly lower resistance to tumor development than those injected with IC_50_ PDT-treated cells. The results presented in this study suggest that both the photosensitizer concentration and light dose affect the immunogenicity of the PDT-treated cells. This event can affect the therapy outcomes in vivo.

## 1. Introduction

Photodynamic therapy (PDT) is generally based on three harmless components: molecular oxygen, photosensitizer, and light [1]. Once combined, they yield a robust production of reactive oxygen species (ROS), which can be lethal to the target cell [2,3,4]. As a cancer treatment modality, PDT can directly kill tumor cells due to its photocytotoxicity, cause infarction of the cancerous tissue by its effects on the tumor microvasculature and activate the immune system against tumor antigens [5]. The activation of the immune system by PDT has been the subject of intense research in recent years. In a murine experimental model of ectopic 4T1 mammary adenocarcinoma, PDT reduced the incidence of metastatic foci in the lungs, even when applied to the primary tumor [6]. Importantly, different parameters of PDT can affect its ability to induce antitumor responses, such as the type [5] and concentration of photosensitizer, as well as the irradiation regimen [7], a fact that has to be taken into account in the design of PDT-based immunotherapy.

The cell death mechanism triggered by PDT seems to play a key role on its immune effects. For instance, PDT mediated by the photosensitizer aluminum-phthalocyanine (AlPc) can induce both necrosis and apoptosis in murine melanoma B16F10 cells [7]. In this PDT setting, necrosis predominates at higher concentrations of AlPc, while apoptosis is the main cell death mechanism elicited at lower concentrations of this photosensitizer [7]. In the context of antitumor immune responses induced by PDT, the induction of immunogenic cell death (ICD) in target cells is to be cited [8]. According to Garg et al. [5], ICD is an apoptosis modality distinguished by the emission of DAMPs, which are potent immune activators. As agonists of various receptors involved in the immune response, DAMPs can attract and activate different immune cells [9]. They are also capable of promoting proinflammatory events, such as the maturation and activation of antigen-presenting cells, such as dendritic cells and T-cell activating macrophages [8,10].

In this study, the immunogenicity of two murine cancer cell lines–colorectal carcinoma (CT26) and mammary adenocarcinoma (4T1) cells–submitted to different PDT protocols mediated by a nanoemulsion containing aluminum-phthalocyanine (PDT-AlPc-NE) was evaluated. AlPc was chosen as the model photosensitizer because of its high singlet oxygen photogeneration yield and for its efficacy against both primary tumors and metastasis of murine cancer cells, such as 4T1 cells [1,6], in in vivo models. However, the possible immune-related, cellular mechanisms behind the efficacy of AlPc have not been studied in those models. The results show that both CT26 and 4T1 cells emitted different DAMP (calreticulin-CRT, heat shock proteins (HSP)-70 and -90, interleukin 1 beta-IL-1B, and high mobility group-box 1 (HMGB1) after specific PDT-AlPc-NE protocols in vitro. In an in vivo vaccination-challenge model, these PDT-treated cells rendered mice more resistant against the development of experimental tumors.

## 2. Materials and Methods

### 2.1. Materials

Roswell Park Memorial Institute medium (RPMI 1640), fetal bovine serum (FBS), penicillin, streptomycin, and trypsin were all purchased from Gibco, Carlsbad, CA, USA. Ethanol (99.3° GL) and glucose were all purchased from J.T. Trypan blue and dimethylsulfoxide (DMSO 99.5%); Trypan blue, 4′,6-diamidino-e-fenilindol (DAPI), Annexin V (AnV), and propidium iodide (PI) were purchased from Sigma-Aldrich (St. Louis, MO, USA). Aluminum-phthalocyanine (AlPc) was purchased from Aldrich Chemical Company (St. Louis, MO, USA). PBS was purchased from Pinhais, Paraná, Brazil. Enzyme-linked immunosorbent assay (ELISA) kit for high mobility group box-1 (HMGB1) was purchased in IBL International GmbH (Hamburg, Germany). ELISA kit for heat shock proteins (HSP)-70 and −90, primary anti-mouse/human antibody against CRT, and Alexa Fluor^®^ 488 Goat Anti-Mouse (IgG) secondary antibodies (IgG488) were purchased from ABCAM (Cambridge, UK). ELISA kit for interleukin-1beta (IL-1β) was purchased from Life Technologies (Carlsbad, CA, USA).

### 2.2. Light Source

A LED array system was used for irradiation of cells in vitro. This system was composed of a 20-LED-lamp-array model XL001WP01NRC660 (Shenzhen Sealand Optoelectronics, Ltd., Shenzhen, Guangdong, China) attached to a metal cooling unit and controlled by a constant current LED driver (Recom Power, Inc., Dietzenbach, Germany) model RCD-24-0.35/W. The energy fluence (J/cm^2^) was adjusted according to the following formula: [power (W) × irradiation time (s)]/area of the light sensor (cm^2^). LED spectral emission was recorded with a portable spectrometer (Ocean Optics, Inc., USA) with a spectral resolution of 0.2 nm in a range of 600–700 nm. A light power meter (Fieldmax II, Coherent Inc., Santa Clara, CA, USA) with a 1.9 cm diameter circular light-sensing area was used to measure maximum power and power as a function of the distance between the illumination system and the target. This system has a maximum power of 800 mW and a maximum fluence rate of 55 mW/cm^2^ inside the 2.5-cm-radius illuminated area. The actual power used was equivalent to 122 mW. The spectral output is limited to the 660 nm red band.

### 2.3. Nanoemulsion Containing Aluminum-Phthalocyanine (AlPc-NE)

The nanoemulsion containing aluminum-phthalocyanine (AlPc-NE) was prepared by the spontaneous nanoemulsification method described by Muehlmann et al. [11]. Briefly, 9 g Kolliphor^®^ ELP and 3 g castor oil were mixed with 40 mL AlPc 100 µM in ethanol. This solution was stirred at 50 °C for 15 min. Ethanol was then removed at 80 °C under magnetic stirring. Next, 70 mL of distilled water was added, and the dispersion was left under stirring at room temperature until a transparent nanoemulsion was obtained. The volume was then completed to 100 mL with PBS. The final concentration of AlPc in this formulation was 40 μM.

### 2.4. Cell Culture

Both the murine colorectal carcinoma (CT26) and the murine mammary adenocarcinoma cell line (4T1) were purchased from American Type Culture Collection (ATCC, Manassas, VA, USA). CT26 cells and 4T1 cells were cultured in RPMI supplemented with 10% (*v*/*v*) FBS, 100 units penicillin/mL, and 100 mg streptomycin/mL and maintained in an incubator under a humidified atmosphere with 5% CO_2_ at 37 °C. The different in vitro tests in this study were performed using either 12- or 96-well microplates, with 4 × 10^4^ CT26 or 1 × 10^4^ 4T1 cells per well.

### 2.5. Animals

Immunocompetent 12-week-old female BALB/c mice were kept in an animal facility, with free access to Purina and water and were handled according to procedures previously approved by the Ethics Committee on Animal Use of the University from Brasilia, Brazil (UnB/Doc n. 5529/2015, approved on 3 March 2015).

### 2.6. PDT-AlPc-NE In Vitro

CT26 or 4T1 cells were incubated in the dark at 37 °C with different concentrations of AlPc-NE for 15 min. Then, the cells were washed with PBS and maintained in an incubator for further 15 min with complete RPMI 1640 medium. Next, the cells were irradiated with LED 660 nm for 10 min, with a final energy dose of 25 J/cm^2^. The AlPc-NE concentrations that reduced cell viability by 50% (IC_50_) and 90% (IC_90_) were then calculated. In subsequent experiments, the cells were treated with PDT protocols, with their respective AlPc-NE IC_50_ and IC_90_, maintained in an incubator and analyzed for cell death pathways and DAMP exposure.

### 2.7. Cell Death Pathways

Two methods were used to verify the cell death pathway triggered in the CT26 cells and 4T1 cells by PDT-AlPc-NE protocols: (i) fluorescence microscopy (microscope EVOS- FL, Thermo Fisher Scientific InC., Waltham, MA, USA) of cells stained with acridine orange and propidium iodide (AO/PI) at 4 h after the PDT-AlPc-NE protocols described by Kasibhatla et al. [12] and (ii) flow cytometry of cells stained with Alexa Fluor 488-annexin V and propidium iodide (AnV/PI) at 24 h after the PDT-AlPc-NE. Mitoxantrone (1.5 µM) was used as a positive control for ICD and apoptosis. The necrosis-positive control group consisted of cells frozen and thawed three times, as described by Garg et al. [13].

### 2.8. Immunofluorescence

The detection of CRT, HSP70, and HSP90 was performed by immunofluorescence. After the treatments, the cells were fixed with ethanol 70% (*v*/*v*, in water) at room temperature for 30 min. Next, the cells were incubated with an anti-CRT (1:75), anti-HSP70 (1:75) or anti-HSP90 (1:150) primary antibody in cold blocking buffer (2% BSA in PBS) for 1 h in an incubator (37 °C), followed by washing with PBS and incubation with IgG488 secondary antibody (1:250) for 30 min at room temperature. The nuclei of the cells were labeled with DAPI. The stained cells were visualized with a fluorescence microscope (EVOS-FL, Thermo Fisher Scientific Inc., Waltham, MA, USA). The fluorescence of the stained cells was quantified with the software Photoshop CC 2015 (Adobe Systems, San Jose, CA, USA) and ImageJ 1.8.0_172 (NIH, Madison, WI, USA).

### 2.9. ELISA

ELISA kits were used to quantify IL-1β (Life Technologies, Carlsbad, CA, USA) and HMGB1 (IBL International GmbH, Hamburg, Germany) in the culture supernatants according to the manufacturer’s protocol 24 h after the difference treatment with PDT-AlPc-NE, MTX, and F-T.

### 2.10. Vaccination-Challenge Assay

The immunogenicity of the treated cells was assessed with an in vivo vaccination-challenge model. Briefly, CT26 cells and 4T1 cells were treated as follows, respectively: (i) PDT1 12.2 nM or 9.01 nM with 25 J/cm^2^; (ii) PDT2 31.5 nM or 19.4 nM with 25 J/cm^2^; (iii) PDT3 12.2 nM or 9.01 nM with 67 J/cm^2^; (iv) PDT4 31.5 nM or 19.4 nM with 67 J/cm^2^; and (v) mitoxantrone (1.5 µM); and (vi) frozen-thawed. Then, 100 µL of the suspension of cells (4 × 10^5^ CT26 or 1 × 10^5^ 4T1 cells/mL) were subcutaneously injected into the right flank of mice. This process was repeated a second time, with a ten-day interval between vaccinations. Seven days after the 2nd vaccine, the mice were challenged with a subcutaneous graft of viable CT26 or 4T1 cells on the left flank. Following the challenge with viable CT26 or 4T1 cells, the animals were monitored for tumor onset, tumor volume evolution, and survival.

### 2.11. Computed Tomography

The mice were anesthetized with ketamine and xylazine (80 and 10 mg/kg, respectively) and subjected to computed tomography (PET-SPECT and CT-Bruker, Ettlingen, Germany). It used low-resolution (CT single good, low dose (200 μA), low voltage (35 μA)), and standard CT quality. Two hundred and fifty image projections were collected per animal with three minutes of exposure. Then, images were reconstructed with standard software reconstruction modes to evaluate the pulmonary radiopacity profile and bone structure. The pulmonary radiopacity profile was assessed in all planes of the image by delimiting the pulmonary region using the inner side of the rib cage as a reference. The software generates an automatic Hounsfield (HU) scale, and the voxel (3D pixel) intensity values create a Gaussian distribution in this HU scale. The less-dense voxels on the scale are positioned on the left, while the denser ones are placed on the right. Increased pulmonary density, which is correlated with lung metastasis [6], was evaluated by the Gaussian curve displacement to the right. The area under the curve of each experimental animal was calculated as the integral of the curve in a bar graph.

### 2.12. Statistical Analyses

All statistical analyses were performed with GraphPad Prism 7.0 software (San Diego, CA, USA). Correlation between variables was analyzed with the Pearson and Spearman test. Significant differences between groups were assessed by one-way analysis of variance (ANOVA) followed by Tukey or Bonferroni’s post-tests (α = 0.05). Results are expressed as mean ± standard error of the mean.

## 3. Results and Discussion

Several studies have shown that PDT is capable of triggering immune responses against tumor antigens [5,14,15]. One of the candidate mechanisms underlying this immune effect is the occurrence of ICD in cancer cells exposed to PDT [5,7]. Thus, the main goal of the present work was to verify whether two critical variables of PDT protocols, namely photosensitizer concentration, and light dose, affect the ability of this approach to induce ICD in two different murine cancer cell lines, 4T1 and CT26, in vitro. CT26 and 4T1 cells were subjected to different PDT-AlPc-NE protocols to obtain IC50 and IC90. The concentrations obtained for CT26 can be found in the Supplementary Materials (Figures S1 and S2). The data for 4T1 cells were published in Rodrigues et al. [1].

**Figure 1:**
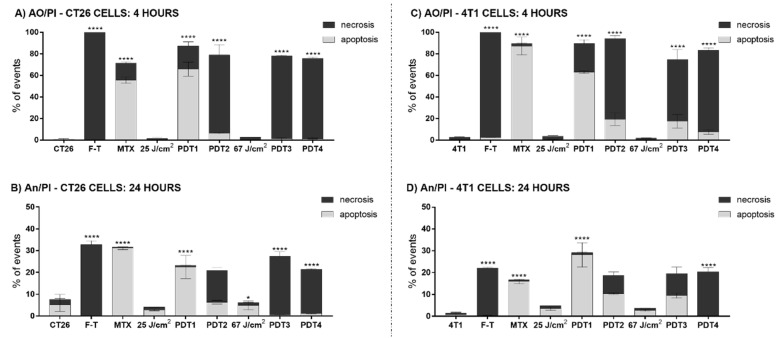
The induction of necrosis and apoptosis by PDT-AlPc-NE is affected by both the concentration of photosensitizer and the energy dose. (**A**) CT26 cells analyzed by the AO/PI method after 4 h of treatment; (**B**) CT26 cells analyzed by AnV/PI method after 24 h of treatments; (**C**) 4T1 cells analyzed by the AO/PI method after 4 h of treatment, and (**D**) 4T1 cells analyzed by the AnV/PI method after 24 h of treatments. Light gray bars represent the results of apoptotic cells, and dark gray bars represent necrotic cells. Untreated cells are represented with CT26 and 4T1.PDT protocols for CT26 cells: PDT1 = 12.2 nM and 25 J/cm^2^; PDT2 = 31.5 nM and 25 J/cm^2^; PDT3 = 12.2 nM and 67 J/cm^2^; and PDT4 = 31.5 nM and 67 J/cm^2^. PDT protocols for 4T1 cells: PDT1 = 9.01 nM and 25 J/cm^2^; PDT2 = 19.4 nM and 25 J/cm^2^; PDT3 = 9.01 nM and 67 J/cm^2^; and PDT4 = 19.4 nM and 67 J/cm^2^. MTX: mitoxantrone; F-T: three cycles of freeze-thawing; AO/PI: acridine orange and propidium iodide; AnV/PI: Annexin V and propidium iodide. Superscripts * and **** represent *p* < 0.05 and *p* < 0.0001 relatives to apoptotic and necrotic cells of the same group. Data are presented as mean ± SEM for triplicates.

ICD is characterized by cell death by apoptosis with a well-defined pattern of DAMP exposure. It is well described that PDT can preferentially trigger apoptosis or necrosis depending on protocol parameters such as the concentration of photosensitizer and the energy dose applied [1,7]. The results in Figure 1 corroborate findings in the literature, as the cell mechanism triggered by PDT depended on the protocol parameters. Both CT26 and 4T1 cells succumbed to apoptosis when submitted to PDT with their respective AlPc-NE IC_50_, 12.2 nM, and 9.01 nM, respectively, and the same energy dose—25 J/cm^2^. As expected, with the same AlPc-NE concentrations, but under a higher energy dose—67 J/cm^2^, a significant increase in the percentage of necrotic cells was observed. Moreover, necrosis was predominant in both CT26 and 4T1 cells exposed to their respective AlPc-NE IC_90_–31.5 and 19.4 nM–at both 25 J/cm^2^ and 67 J/cm^2^ energy doses (Figure 1).

It is noteworthy that the results profiles observed for both cell lines treated with IC_50_ and 25 J/cm^2^ were similar to those obtained with the ICD-positive control MTX. As expected, F-T induced necrosis in both cells studied. The treatments with MTX and F-T are often used as positive controls for apoptosis and necrosis, respectively [7,13]. Thus, variations in the PDT-AlPc-NE protocol parameters affect the type of cell death induced in the studied cells, which can be correlated to the intensity of the oxidative stress in the target cell following PDT.

**Figure 2:**
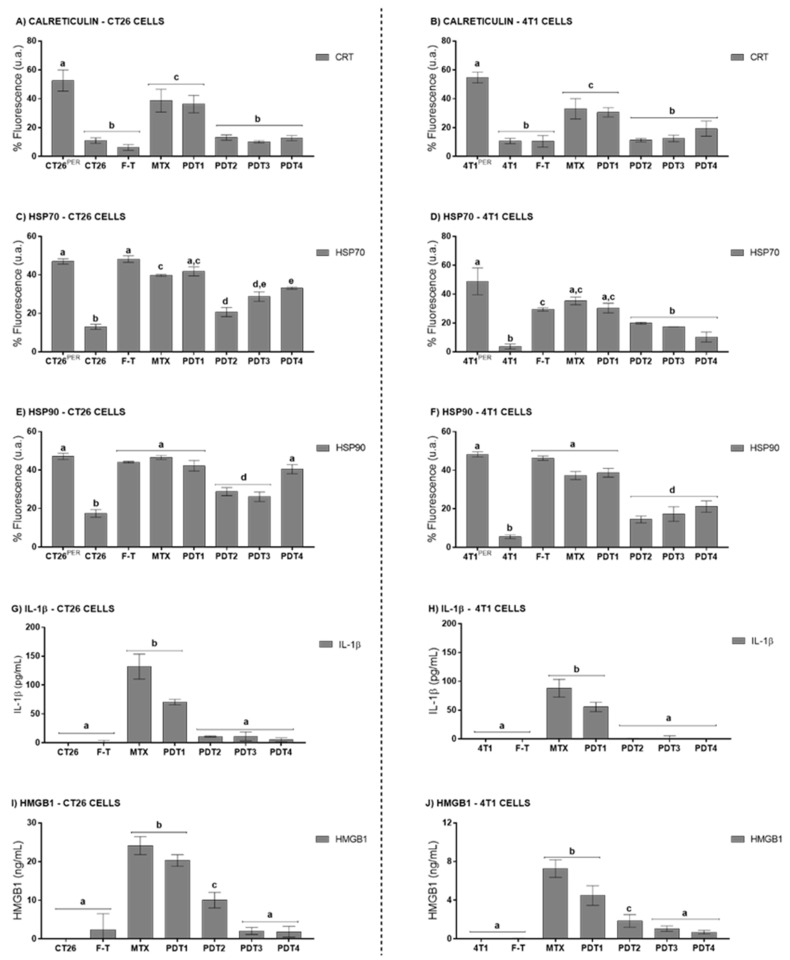
PDT-AlPc-NE induces the release of DAMPs by murine colorectal carcinoma (CT26) and murine mammary adenocarcinoma (4T1) cells. Surface CRT (**A**,**B**), surface HSP70 (**C**,**D**), and surface HSP90 (**E**,**F**). Supernatant IL-1β (**G**,**H**) and HMGB1 (**I**,**J**) letters refer to CT26 and 4T1 cells, respectively. Untreated cells are representing with CT26 and 4T1.PDT protocols for CT26 cells: PDT1 = 12.2 nM and 25 J/cm^2^; PDT2 = 31.5 nM and 25 J/cm^2^; PDT3 = 12.2 nM and 67 J/cm^2^; and PDT4 = 31.5 nM and 67 J/cm^2^. PDT protocols for 4T1 cells: PDT1 = 9.01 nM and 25 J/cm^2^; PDT2 = 19.4 nM and 25 J/cm^2^; PDT3 = 9.01 nM and 67 J/cm^2^; and PDT4 = 19.4 nM and 67 J/cm^2^. MTX: mitoxantrone; F-T: three cycles of freeze-thawing. Superscripts ^PER^ mean the cells submitted to permeation with Triton X-100 0.1%. Equal letters represent results without significant differences between groups. Data are presented as mean ± SEM for triplicates.

The profile of DAMPs released by cells succumbing to PDT was also investigated. The exposure of CRT on the plasma membrane is an essential feature of ICD, as this DAMP facilitates the recognition and phagocytosis of the target cell by antigen-presenting cells [13,16,17]. These, in turn, will present the processed tumor cell antigens, potentially inducing an antitumor immune response mediated by CD8^+^ T cells [18,19]. Moreover, the exposure of HSP70 and HSP90 can increase the immunogenicity of the cells [20,21]. As shown in Figure 2, the exposure of HSP70, HSP90, and CRT were affected by variations in PDT-NE-AlFtCl parameters in both CT26 and 4T1 cells. The images used for fluorescence quantification can be found in the Supplementary Materials (Figures S3–S5). When these cells were submitted to PDT with AlPc-NE IC_50_ and an energy dose of 25 J/cm^2^, a more intense CRT exposure, HSP70, and HSP90 was observed. A significantly lower exposure of these DAMPs was observed with the PDT protocols based on higher AlPc concentrations and higher energy doses. Other studies have also shown this same dose-dependency concerning DAMP exposure [22,23]. As expected, MTX induced HSP70, HSP90, and CRT exposure, while the F-T process did not cause the exposure of these DAMPs on the plasma membrane. The apparent increased exposure of HSP70 and HSP90 in CT26 cells treated with PDT4 is most probably due to the disruption of the plasma membrane that occurs in necrotic cells, which enables for the staining of these intracellular proteins by immunofluorescence.

Another important hallmark of ICD is the release of HMGB1 to the extracellular medium [24,25,26]. HMGB1 is a nuclear protein associated with nucleosomes [26]. HMGB1 protein is released during the late phase of ICD, since long after the onset of apoptosis, and the chromatin becomes deconcentrated and, consequently, HMGB1 release occurs [24,26,27]. This DAMP attracts DC cells and macrophages; upon recognition, these cells become mature and responsible for activating T cells [13,28]. The results presented in Figure 2 show that all the tested PDT protocols induced the release of HMGB1 by both CT26 and 4T1 cells, with a more intense release being observed with the protocol with AlPc IC_50_ and energy dose of 25 J/cm^2^.

The release of the proinflammatory cytokine IL-1β was also assessed. For both the 4T1 and CT26 lines, only PDT with AlPc IC_50_ and 25 J/cm^2^, and MTX, promoted a significant release of IL-1β compared to control (Figure 2G). IL-1β can modify the activity of many immune cell types, such as monocytes, macrophages, neutrophils, and lymphocytes, and can induce the release of other essential cytokines and chemokines involved in the activation of adaptive immune responses [29,30].

Thus, the in vitro experiments suggest that PDT-AlPc-NE can induce apoptosis and DAMPs exposure, a death pattern characteristic of ICD, in CT26 cells and 4T1 cells. Moreover, the results evidence that both the percentage of apoptotic cells and the DAMPs release profile are affected by the PDT parameters, specifically the AlPc-NE concentration and the energy dose.

**Figure 3:**
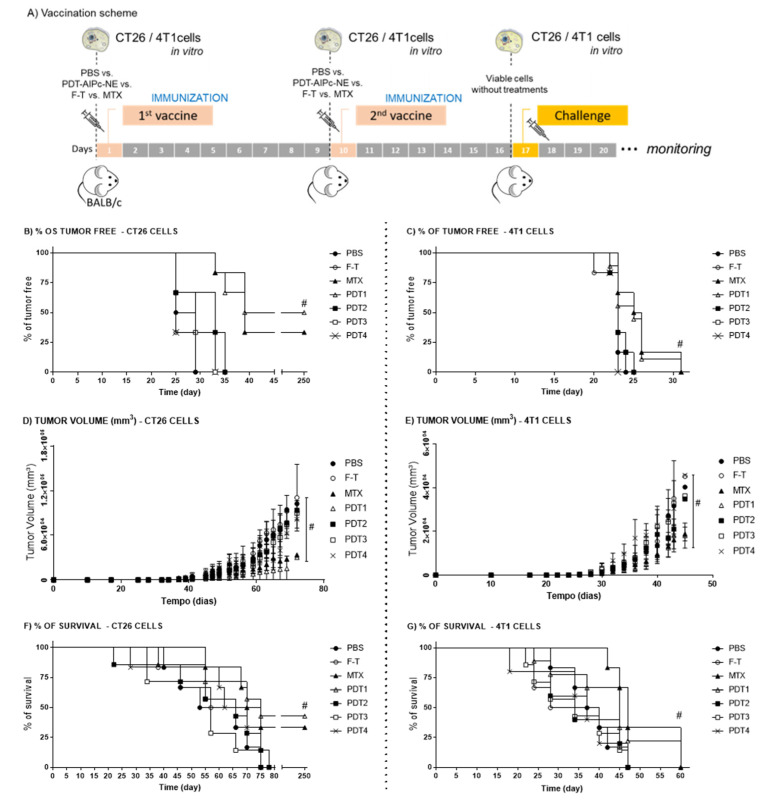
Assessment of the in vivo immunogenicity of cells treated with different protocols of photodynamic therapy. (**A**) Representation of the vaccination-challenge schedule using CT26 cells and 4T1 cells treated with different PDT-AlPc-NE protocols vs F-T vs MTX. CNTR represent the animal that received just PBS without cells. After the challenge the animals were monitored for: the onset of tumors (**B**,**C**); tumor volume (**D**,**E**); and survival (**F**,**G**) referring CT26 and 4T1, respectively. PDT protocols for CT26 cells—: PDT1 = 12.2 nM and 25 J/cm^2^; PDT2 = 31.5 nM and 25 J/cm^2^; PDT3 = 12.2 nM and 67 J/cm^2^; and PDT4 = 31.5 nM and 67 J/cm^2^. PDT protocols for 4T1 cells: PDT1 = 9.01 nM and 25 J/cm^2^; PDT2 = 19.4 nM and 25 J/cm^2^; PDT3 = 9.01 nM and 67 J/cm^2^; and PDT4 = 19.4 nM and 67 J/cm^2^. 1st vaccine day 0; 2nd vaccine day 10 and the challenge day 17. MTX (mitoxantrone); F-T (three cycles of freeze-thawing); PBS: phosphate buffered saline. Superscript # means *p* < 0.05 in comparison to control group (PBS) at the endpoint. For all data, *n* = 6 mice, mean ± SEM.

As suggested by the literature [31], the immunogenicity of cells undergoing ICD can be assessed in in vivo vaccination-challenge models. Thus, CT26 cells and 4T1 cells subjected to different in vitro PDT protocols were used as prophylactic vaccines injected subcutaneously into the flank of the animals, as shown in Figure 3. The results show that 50% and 40% of mice vaccinated with CT26 cells treated with PDT1 [12.2 nM and 25 J/cm^2^] and MTX, respectively, did not develop tumors up to 250 days after the challenge (Figure 3B). This result further shows that PDT can elicit ICD, an event already described in the literature. According to Garg et al. [21], 70% of mice vaccinated with CT26 cells treated with hypericin-mediated PDT were tumor-free.

Interestingly, although 4T1 cells exhibited an ICD-related pattern of DAMPs exposure in the in vitro tests described above, they were less immunogenic in vivo than the CT26 cells (Figure 3C). The 4T1 cells treated with PDT or MTX did not elicit a fully protective immunization of the mice; all the animals presented tumors during the experiment. However, there was a significant delay in tumor development in animals vaccinated with MTX- or PDT1-treated cells compared to the other groups (Figure 3C,D). Moreover, these mice showed a prolonged survival time (Figure 3F).

The CT26 and 4T1 cells have distinct characteristics, such as their origin, immunogenic profile, and dissemination pattern. The 4T1 murine cells come from a spontaneously originated stage IV breast adenocarcinoma and exhibit a high metastization potential to the lungs, bones, liver, spleen, lymph nodes, and brain [6,32,33]. The CT26 murine cell line is derived from a chemically induced tumor, experimentally developed by the administration of N-nitrous-N-methylurethane [34]. Although CT26 cells can generate lung metastasis, they are less aggressive than the 4T1 cells, which can be a consequence of differences in the efficacy of their respective immunoevasion strategies [6,35,36]. The literature suggests that CT26 cells are highly immunogenic [37].

Phenotypically, the 4T1 cells can create an immune-suppressive environment that avoids T cell surveillance, thus protecting tumor cells [6]. The mechanisms are related to the abnormal hematopoiesis process, which is driven to the myeloid lineage that produces more myeloid cells in this unusual event. Moreover, due to an excess of growth factors produced by the 4T1 cells, the produced cells are primarily immature cells with immunosuppressive activity. The consequence of this process is an imbalance between the collective memory lymphocytic response, triggered by the vaccination, and the immunosuppressive actions coordinated by the immature immunosuppressive myeloid cells generated after the 4T1 cells stimuli. All these immunological conditions created by the 4T1 cells can explain why the vaccination is less effective in this model compared to the CT26 tumor model.

**Figure 4:**
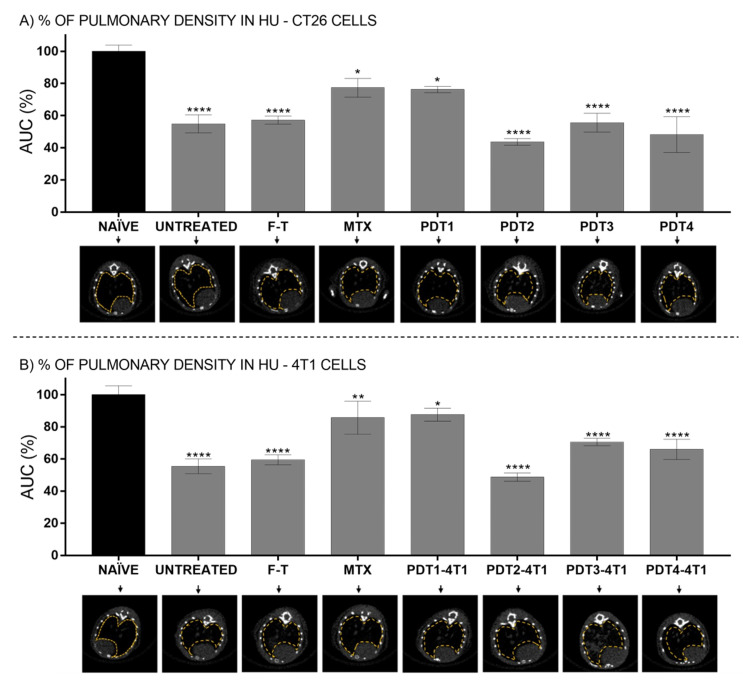
In vivo CT quantification of lung density area using HU values. (**A**) Lung density of animals subjected to vaccination with CT26 cells pretreated with PBS (untreated); F-T; MTX and different PDT protocols. (**B**) Lung density of animals subjected to vaccination with 4T1 cells pretreated with PBS (untreated); F-T; MTX and different PDT protocols. Group of healthy animals (naïve) were used as the control for evaluation of lung density (100%-black bars). Lung 2D representative CT-transverse of the animals references the groups: CT26 cells—PDT protocols: PDT1 = 12.2 nM and 25 J/cm^2^; PDT2 = 31.5 nM and 25 J/cm^2^; PDT3 = 12.2 nM and 67 J/cm^2^; and PDT4 = 31.5 nM and 67 J/cm^2^. 4T1 cells—PDT protocols: PDT1 = 9.01 nM and 25 J/cm^2^; PDT2 = 19.4 nM and 25 J/cm^2^; PDT3 = 9.01 nM and 67 J/cm^2^; and PDT4 = 19.4 nM and 67 J/cm^2^. MTX (mitoxantrone); F-T (three cycles of freeze-thawing); PBS: phosphate buffered saline. Superscript *, ** and **** means *p* < 0.05, *p* < 0.01 and *p* < 0.001, respectively. For all data, *n* = 6 mice, mean ± SEM.

This study also evaluated the appearance of metastasis foci in the lungs of animals submitted to vaccines with CT26 or 4T1 cells (Figure 4 and Supplementary Materials Figures S6 and S7, respectively). It was found that animals vaccinated with CT26 cells treated with PDT1 had a lung density similar to that of healthy animals, suggesting a low incidence of metastasis (Figure 4). It has already been shown a reduction in CT26 cells metastatic foci in the lungs of mice treated with bacteriochlorin-mediated PDT, an event associated with an immune response induced by PDT against these cells [38]. Regarding the 4T1 cells, a high incidence of metastasis was found in tumor-bearing control mice. However, the radiopacity of the lungs of animals vaccinated with cells treated with PDT1 or MTX was not statistically different from that presented by control, healthy animals (Figure 4B). This result further suggests that, even though these mice (PDT1 and MTX) presented tumors, as discussed earlier, the development of both the grafted tumor and the metastatic foci was somehow reduced. This could be due to an immune response induced in mice by the 4T1 cells succumbing to ICD. Previously, Longo et al. [6] showed that PDT significantly prolonged the survival of mice bearing grafted 4T1 cells tumor, an event associated with a drastic reduction in the number of metastatic foci in the lungs. Moreover, the authors showed that PDT reduced the count of myeloid-derived suppressor cells (MDSC) in the spleen, which can be linked to a reduced ability of 4T1 cells to escape the immunosurveillance and to establish metastasis in PDT-treated mice [6]. That finding could result from the PDT-induced ICD in 4T1 cells, affecting the systemic immune response against tumor cells.

## 4. Conclusions

The present study suggests that the concentration of photosensitizer and the energy dose are important parameters regarding the ability of PDT to induce ICD in CT26 and 4T1 cells. The application of a milder PDT-AlPc-NE rendered the cells more immunogenic than the more intense PDT protocols. Further studies must address how variations on PDT parameters can affect the in vivo activation of the immune system.

## Data Availability

Data is contained within the article or supplementary material.

## Supplementary Materials

The following are available online at www.mdpi.com/article/10.3390/pharmaceutics14010196/s1, Figure S1: Figure S1. Photodynamic effect of AlPc-NE in CT26 cells. The black line represents the CT26 cells exposed to AlPc-NE and maintained in the dark (non-irradiated). The gray line represents the viability of CT26 cells exposed to AlPc-NE for 15 min, washed, left in the dark for different times: (A) 0; (B) 15; (C) 30; (D) 45; (E) 105; and (F) 225 min, respectively. After the incubation, the cells were irradiated for 10 min with a light-emitting diode (LED, λ 660 nm, final energy density of 25 J/cm^2^). IC_50_: inhibitory concentrations 50%; IC_90_: inhibitory concentrations 90%. Data are presented as mean ± SEM for triplicates. Figure S2: Cell viability as a function of the energy density applied (LED, λ 660 nm). The cells were exposed to AlPc IC_50_ and irradiated at the specific incubation-to-irradiation times (LED, λ 660 nm) as follows: (A) 0; (B) 15; (C) 30; (D) 45; (E) 105; and (F) 225 min, respectively. Subscript ^#^ *p* < 0.01 vs control (100%). Data are presented as mean ± SEM for triplicates. Figure S3. Exposure of calreticulin by CT26 and 4T1 cells exposed to different treatments in vitro. The results for CT26 cells are shown in (A–I) (left panel), and for 4T1 cells in (J–R) (right panel). (A) and (J): Triton X-100 permeabilized cells (TX100); (B) and (K): unpermeabilized control cells; (C) and (L): MTX-treated cells (1.5 µM); (D) and (M): cells subjected to F-T; (E) and (N): PDT1; (F) and (O): PDT2; (G) and (P): PDT3; (H) and (Q): PDT4; (I) and (R): All results for the DAPI marking profile for blue nucleus with green CRT are shown. Data plotted as ± SEM for triplicates. Figure S4. Exposure of HSP70 by CT26 and 4T1 cells exposed to different treatments in vitro. The results for CT26 cells are shown in (A–I) (left panel), and for 4T1 cells in (J–R) (right panel). (A) and (J): Triton X-100 permeabilized cells (TX100); (B) and (K): unpermeabilized control cells; (C) and (L): MTX-treated cells (1.5 µM); (D) and (M): cells subjected to F-T; (E) and (N): PDT1; (F) and (O): PDT2; (G) and (P): PDT3; (H) and (Q): PDT4; (I) and (R): All results for the DAPI marking profile for blue nucleus with green HSP70 are shown. Data plotted as ± SEM for triplicates. Figure S5. Exposure of HSP90 by CT26 and 4T1 cells exposed to different treatments in vitro. The results for CT26 cells are shown in (A–I) (left panel), and for 4T1 cells in (J–R) (right panel). (A) and (J): Triton X-100 permeabilized cells (TX100); (B) and (K): unpermeabilized control cells; (C) and (L): MTX-treated cells (1.5 µM); (D) and (M): cells subjected to F-T; (E) and (N): PDT1; (F) and (O): PDT2; (G) and (P): PDT3; (H) and (Q): PDT4; (I) and (R): All results for the DAPI marking profile for blue nucleus with green HSP90 are shown. Data plotted as ± SEM for triplicates. Figure S6: In vivo computed tomography quantification of the frequency of voxel as a function of HU in the lung. Lung density of animals subjected to vaccination with CT26 cells pretreated: (A) NAÏVE ANIMALS and (A.1) results showed as AUC; (B) PBS (untreated) and (B.1) results showed as AUC AUC; (C) F-T and (C.1) results showed as AUC; (D) MTX and (D.1) results showed as AUC; (E to H) CT26 cells – PDT protocols: PDT1 = 12.2 nM and 25 J/cm^2^; PDT2 = 31.5 nM and 25 J/cm^2^; PDT3 = 12.2 nM and 67 J/cm^2^; and PDT4 = 31.5 nM and 67 J/cm^2^ and E.1 to H.1) results showed as AUC, respectively. The assays were performed with a difference of one week (assay 1 to assay 2). For all data, *n* = 6 mice, mean ± SEM. Figure S7. In vivo computed tomography quantification of the frequency of voxel as a function of HU in the lung. Lung density of animals subjected to vaccination with 4T1 cells pretreated: (A) NAÏVE ANIMALS and (A.1) results showed as AUC; (B) PBS (untreated) and (B.1) results showed as AUC AUC; (C) F-T and (C.1) results showed as AUC; (D) MTX and (D.1) results showed as AUC; (E to H) 4T1 cells – PDT protocols: PDT1 = 9.01 nM and 25 J/cm^2^; PDT2 = 19.4 nM and 25 J/cm^2^; PDT3 = 9,01 nM and 67 J/cm^2^; and PDT4 = 19.4 nM and 67 J/cm^2^ and E.1 to H.1) results showed as AUC, respectively. The assays were performed with a difference of one week (assay 1 to assay 2). For all data, *n* = 6 mice, mean ± SEM.
