# Supplementary material for: Induction of Immunogenic Cell Death by Photodynamic Therapy Mediated by Aluminum-Phthalocyanine in Nanoemulsion
Source: Pharmaceutics. 2022 Jan 14;14(1):196. doi: 10.3390/pharmaceutics14010196 (PMC8778058; doi:10.3390/pharmaceutics14010196)
Supplement: Supplementary file 1 [file pharmaceutics-14-00196-s001.zip › pharmaceutics-1516960-supplementary.pdf]

pharmaceutics

Supplementary Materials: Induction of Immunogenic Cell Death by Photodynamic Therapy Mediated by Aluminum-Phthalocyanine in Nanoemulsion

Mosar Corrêa Rodrigues, Wellington Tavares de Sousa Júnior, Thayná Mundim, Camilla Lepesqueur Costa Vale, Jaqueline Vaz de Oliveira, Rayane Ganassin, Thyago José Arruda Pacheco, José Athayde Vasconcelos Morais, João Paulo Figueiró Longo, Ricardo Bentes Azevedo and Luis Alexandre Muehlmann

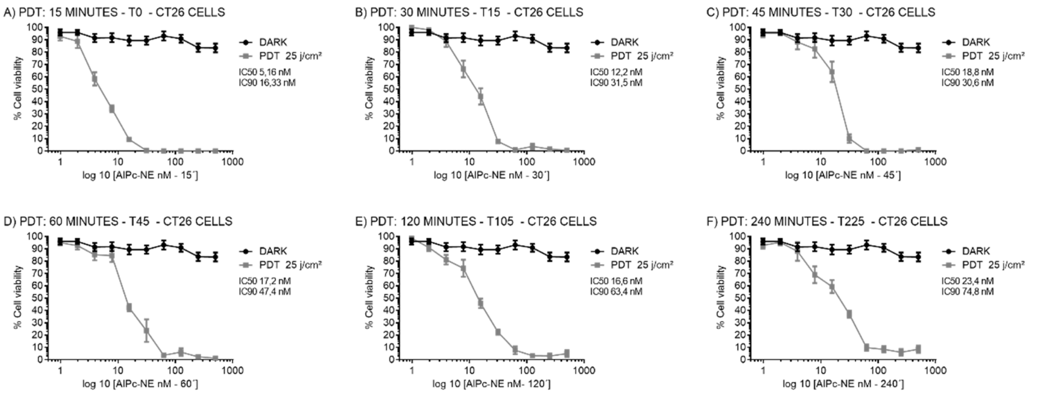

Figure S1. Photodynamic effect of AlPc-NE in CT26 cells. The black line represents the CT26 cells exposed to AlPc-NE and maintained in the dark (non-irradiated). The gray line represents the viability of CT26 cells exposed to AlPc-NE for 15 minutes, washed, left in the dark for different times: (**A**) 0; (**B**) 15; (**C**) 30; (**D**) 45; (**E**) 105; and (**F**) 225 min, respectively. After the incubation, the cells were irradiated for 10 min with a light-emitting diode (LED, λ 660 nm, final energy density of 25 J/cm^2). IC_{50}: inhibitory concentrations 50%; IC_{90}: inhibitory concentrations 90%. Data are presented as mean ± SEM for triplicates.

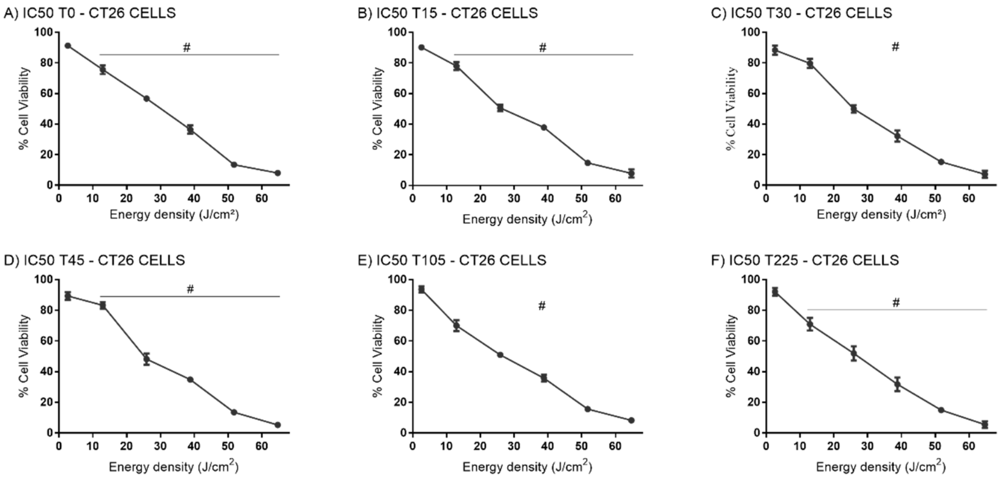

Figure S2. Cell viability as a function of the energy density applied (LED, λ 660 nm). The cells were exposed to AlPc IC_{50} and irradiated at the specific incubation-to-irradiation times (LED, λ 660 nm) as follows: (**A**) 0; (**B**) 15; (**C**) 30; (**D**) 45; (**E**) 105; and (**F**) 225 min, respectively. Subscript $^{\#}$ $p < 0.01$ vs control (100%). Data are presented as mean ± SEM for triplicates.

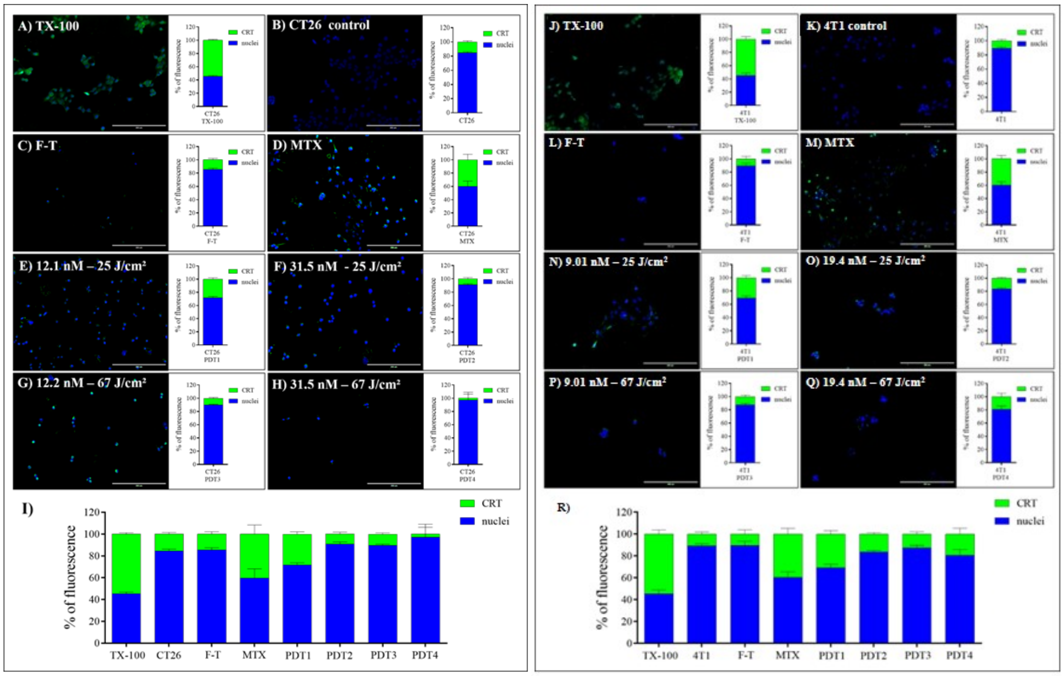

Figure S3. Exposure of calreticulin by CT26 and 4T1 cells exposed to different treatments in vitro. The results for CT26 cells are shown in (**A**–**I**) (left panel), and for 4T1 cells in (**J**–**R**) (right panel). (**A**) and (**J**): Triton X-100 permeabilized cells (TX100); (**B**) and (**K**): unpermeabilized control cells; (**C**) and (**L**): MTX-treated cells (1.5 μM); (**D**) and (**M**): cells subjected to F-T; (**E**) and (**N**): PDT1; (**F**) and (**O**): PDT2; (**G**) and (**P**): PDT3; (**H**) and (**Q**): PDT4; (**I**) and (**R**): All results for the DAPI marking profile for blue nucleus with green CRT are shown. Data plotted as ± SEM for triplicates.

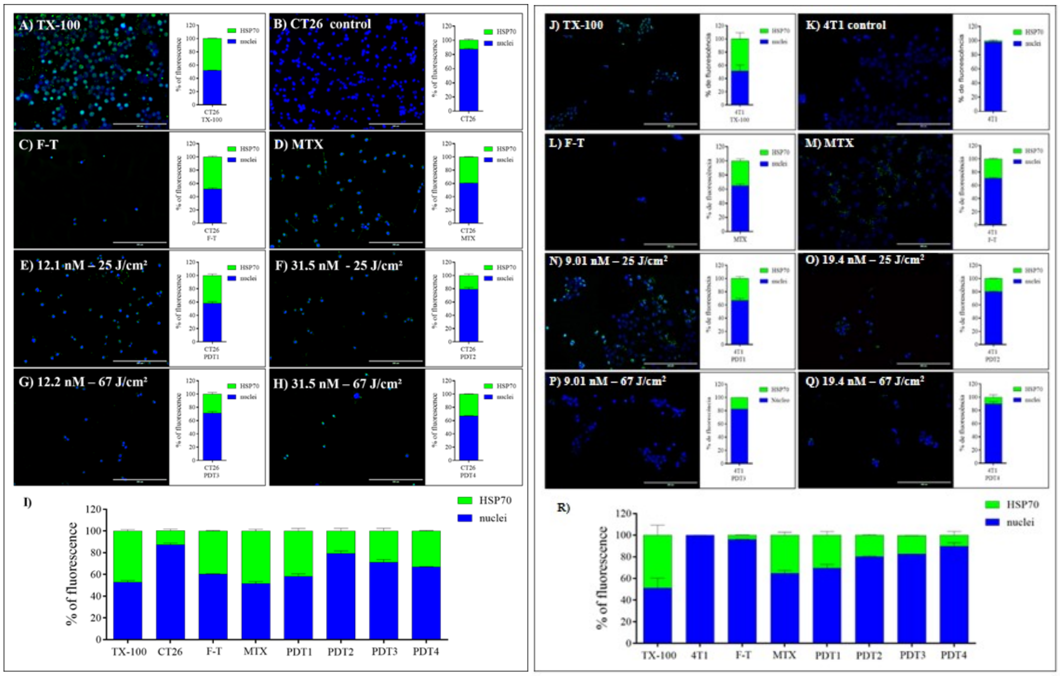

Figure S4. Exposure of HSP70 by CT26 and 4T1 cells exposed to different treatments in vitro. The results for CT26 cells are shown in (**A**–**I**) (left panel), and for 4T1 cells in (**J**–**R**) (right panel). (**A**) and (**J**): Triton X-100 permeabilized cells (TX100); (**B**) and (**K**): unpermeabilized control cells; (**C**) and (**L**): MTX-treated cells (1.5 μM); (**D**) and (**M**): cells subjected to F-T; (**E**) and (**N**): PDT1; (**F**) and (**O**): PDT2; (**G**) and (**P**): PDT3; (**H**) and (**Q**): PDT4; (**I**) and (**R**): All results for the DAPI marking profile for blue nucleus with green HSP70 are shown. Data plotted as ± SEM for triplicates.

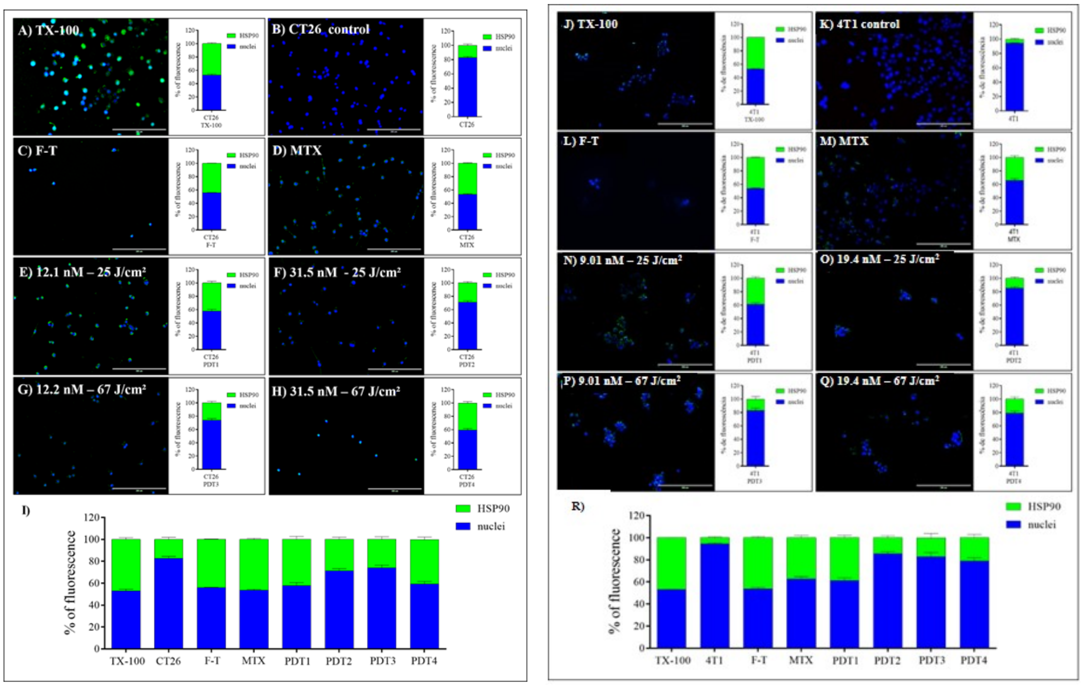

Figure S5. Exposure of HSP90 by CT26 and 4T1 cells exposed to different treatments in vitro. The results for CT26 cells are shown in (**A**–**I**) (left panel), and for 4T1 cells in (**J**–**R**) (right panel). (**A**) and (**J**): Triton X-100 permeabilized cells (TX100); (**B**) and (**K**): unpermeabilized control cells; (**C**) and (**L**): MTX-treated cells (1.5 μM); (**D**) and (**M**): cells subjected to F-T; (**E**) and (**N**): PDT1; (**F**) and (**O**): PDT2; (**G**) and (**P**): PDT3; (**H**) and (**Q**): PDT4; (**I**) and (**R**): All results for the DAPI marking profile for blue nucleus with green HSP90 are shown. Data plotted as ± SEM for triplicates.

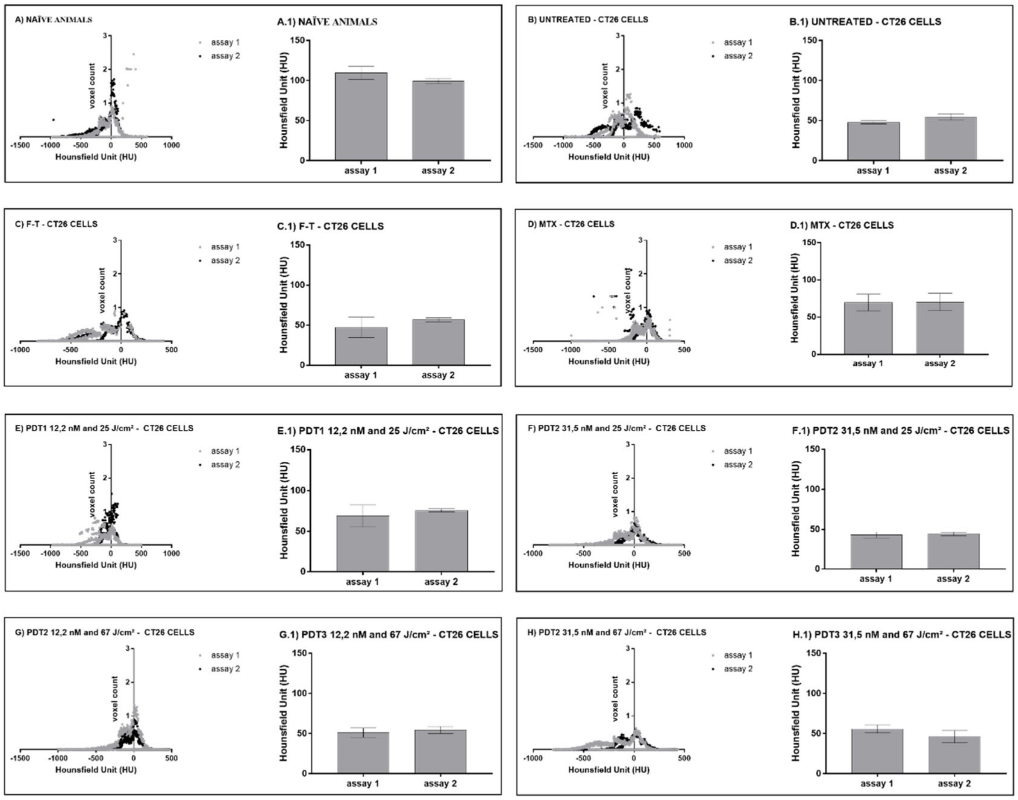

Figure S6. In vivo computed tomography quantification of the frequency of voxel as a function of HU in the lung. Lung density of animals subjected to vaccination with CT26 cells pretreated: (**A**) NAÏVE ANIMALS and (**A.1**) results showed as AUC; (**B**) PBS (untreated) and (**B.1**) results showed as AUC AUC; (**C**) F-T and (**C.1**) results showed as AUC; (**D**) MTX and (**D.1**) results showed as AUC; (**E** to **H**) CT26 cells – PDT protocols: PDT1 = 12.2 nM and 25 J/cm²; PDT2 = 31.5 nM and 25 J/cm²; PDT3 = 12.2 nM and 67 J/cm²; and PDT4 = 31.5 nM and 67 J/cm² and **E.1** to **H.1**) results showed as AUC, respectively. The assays were performed with a difference of one week (assay 1 to assay 2). For all data, *n* = 6 mice, mean ± SEM.

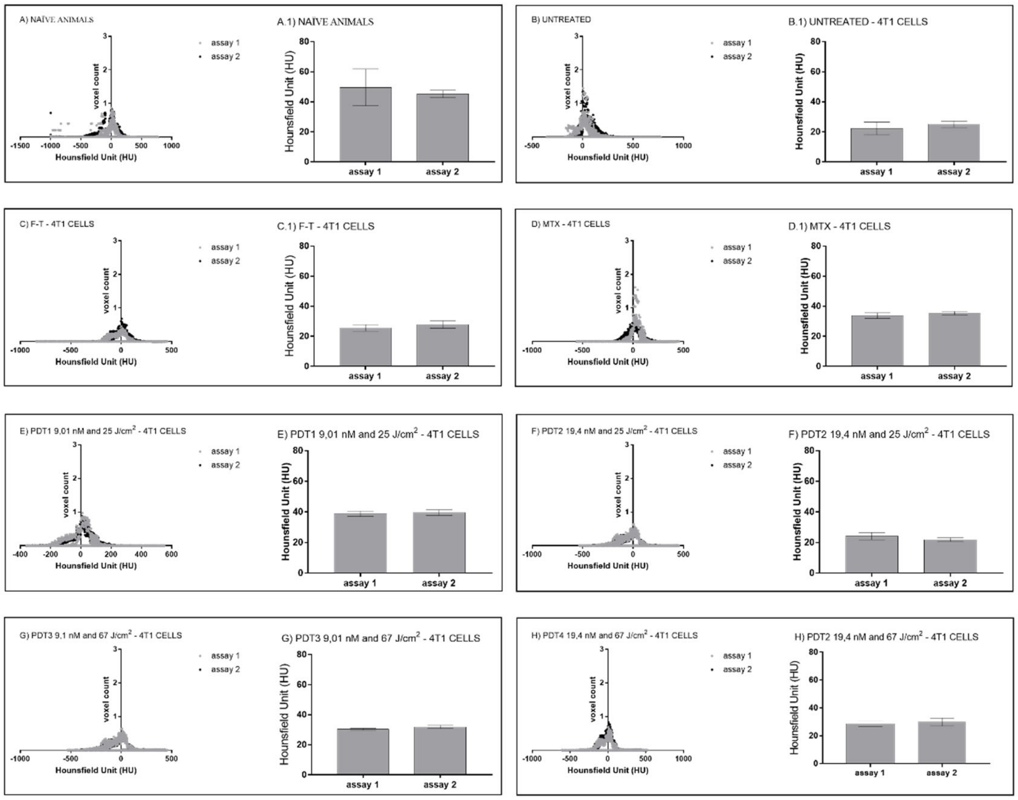

Figure S7. In vivo computed tomography quantification of the frequency of voxel as a function of HU in the lung. Lung density of animals subjected to vaccination with 4T1 cells pretreated: (**A**) NAÏVE ANIMALS and (**A.1**) results showed as AUC; (**B**) PBS (untreated) and (**B.1**) results showed as AUC AUC; (**C**) F-T and (**C.1**) results showed as AUC; (**D**) MTX and (**D.1**) results showed as AUC; (**E** to **H**) 4T1 cells – PDT protocols: PDT1 = 9.01 nM and 25 J/cm^2; PDT2 = 19.4 nM and 25 J/cm^2; PDT3 = 9,01 nM and 67 J/cm^2; and PDT4 = 19,4 nM and 67 J/cm^2 and **E.1** to **H.1**) results showed as AUC, respectively. The assays were performed with a difference of one week (assay 1 to assay 2). For all data, *n* = 6 mice, mean ± SEM.
